# Engaging with Comparative Risk Appraisals: Public Views on Policy Priorities for Environmental Risk Governance

**DOI:** 10.1111/risa.12735

**Published:** 2017-03-17

**Authors:** Sophie A. Rocks, Iljana Schubert, Emma Soane, Edgar Black, Rachel Muckle, Judith Petts, George Prpich, Simon J. Pollard

**Affiliations:** ^1^ Cranfield University, School of Water Energy and Environment Cranfield Bedfordshire UK; ^2^ Department of Management London School of Economics London UK; ^3^ Department for Environment Food and Rural Affairs London UK; ^4^ University of Plymouth Office of the Vice‐Chancellor, Drake's Circus Plymouth PL4 8AA United Kingdom

**Keywords:** Environment, policy prioritization, strategic risk

## Abstract

Communicating the rationale for allocating resources to manage policy priorities and their risks is challenging. Here, we demonstrate that environmental risks have diverse attributes and locales in their effects that may drive disproportionate responses among citizens. When 2,065 survey participants deployed summary information and their own understanding to assess 12 policy‐level environmental risks singularly, their assessment differed from a prior expert assessment. However, participants provided rankings similar to those of experts when these same 12 risks were considered as a group, allowing comparison between the different risks. Following this, when individuals were shown the prior expert assessment of this portfolio, they expressed a moderate level of confidence with the combined expert analysis. These are important findings for the comprehension of policy risks that may be subject to augmentation by climate change, their representation alongside other threats within national risk assessments, and interpretations of agency for public risk management by citizens and others.

## 1. INTRODUCTION

In England, environmental risks such as flooding and animal disease that adversely affect public goods have policies set for their management by government. Even with a multitude of interventions being delivered by a range of actors, each with its own responsibility for managing risks down to lower levels of nonzero “residual risk,” an ongoing challenge is to prioritize the use of annual government resources across a diverse risk portfolio. The transparent allocation of resources for portfolio risk management is difficult, especially in a climate when public finances are constrained, as difficult choices must be made. Under these conditions, there is a continual possibility that lesser risks that are high in the public consciousness, political, or media spotlight may be prioritized above those with more severe or lasting impacts. Such a mismatch between severity and funding provision may be a response to political or reputational damage but may also be due to the lack of impact such funding will make, particularly in situations where effective management measures are not possible. In addition, the competing priorities, dynamics of policy developments, and political influences may impact on the funding provision. It is appropriate therefore that we analyze the tensions between expert and lay audience interpretations of comparative risk so that we are aware of potential anisotropies in understanding and can improve the messaging of risk significance and agency among risk managers.

The academic research team in this study has a long‐standing interest in the capability of governments and their agencies to manage environmental policy risk portfolios.^1^, ^2^ The methodological challenges associated with comparing risks that are disparate in character, such as exposures to engineered nanomaterials (ENMs) alongside the dangers of coastal erosion, for example, where the types and scales of impact vary significantly, are understood and reported widely elsewhere.^3^, ^4^, ^5^, ^6^ Most environmental policy risks are now susceptible to augmentation, in terms of their magnitude and future uncertainty, by the effects of climate change^3^ and so their baseline assessment and future dynamic trajectories are of increasing concern as climate effects exacerbate, or mitigate, their probabilities and impacts forward in time. We have previously commented on the utility of a strategic appraisal method to help risk managers compare national environmental risks at the policy level.^2^, ^7^ A central research question remains: Do these comparative analyses have traction with citizens?

Comparative risk appraisals that inform policy priorities^2^ should have traction with stakeholders^8^ so that the investment priorities they inform are legitimate. Given this, understanding the wider perceptions of policy‐level risks that extend beyond the preserve of policy officials and academic researchers is of research interest. The extent to which comparative risk analyses are reconciled, or otherwise, with citizen views is untested to date in the United Kingdom, though of considerable importance given the wide use of policy risk appraisal in, for example, departmental and national risk registers. National risk assessments are not without controversy^9^ and so it is quite appropriate to ask the question: How do citizens comprehend comparative risk analyses?

Here, we present data from an England‐wide survey of 12 environmental policy risks.^10^ Using a web‐based survey instrument, we explored: (i) citizen understandings of an expert‐driven comparative analysis of 12 environmental risks, considered as a portfolio (addressing issues of comprehension); (ii) the “ordering” and comparative mapping of these 12 risks by survey participants; and (iii) citizen views on the various accountabilities of the actors with responsibility for managing these residual risks (exploring issues of accountability, agency, and trust by reference to those sections of society deemed responsible for risk management).

## 2. METHODS

The policy risks for this study were selected with Department for Environment, Food and Rural Affairs’ (Defra) staff to provide a risk portfolio purposefully diverse in character. Twelve risks from Defra's policy portfolio at the time of the study (2012) were selected that had previously been characterized in autumn 2011 for 12–18 months forward in time:^2^, ^7^ poor air quality; the risk of an avian influenza (AI) incursion; the accelerated spread of bovine tuberculosis (BTb); risks from coastal erosion; the risk of regional‐scale flooding; the risk of a foot and mouth disease (FMD) incursion exposure to genetically modified organisms (GMOs); loss of marine biodiversity; exposures to ENMs; human health effects from pesticides; the risk of a derogation of water quality; and the risk of a loss of wildlife biodiversity.

A web‐based survey investigated whether this published comparison of environmental risks^7^ would be understood by citizens (the questions and response scales used in the questionnaire are presented in the Supporting Information). Brief descriptions of these 12 risks were included in the survey to support respondents’ assessments of severity and likelihood, and their comprehension of this information explored. For example, the risks associated with coastal erosion were presented as:

Natural weathering processes — waves, tides, currents and storm surges — constantly affect the English coastline, causing erosion. The coastlines that are most affected are those on the east and south of the country, and those around the Isle of Wight. Erosion is either gradual or drastic (e.g. cliff slump). Local authorities estimate that 200 properties may be lost over the next 20 years, with approximately 2,000 vulnerable properties in England. The threat of coastal erosion reduces property values and has a detrimental impact on individual and community well‐being. Coastal erosion does provide natural benefits to beaches and habitat.

The presented descriptions of the 12 risks are included within the Supporting Information. The impact of each risk was evaluated using six environmental sustainability criteria:^7^ the economic, environmental, and social impacts (each with direct and indirect impacts) for England over the next 12–18 months.

The survey instrument^11^ was developed by a panel of five researchers and refined with input from Defra staff. A total of 2,179 participants were recruited via a marketing organization database (http://www.maximiles.com;; participants received points redeemable against online shopping) between May 22 and June 6, 2012. Participants were screened for British citizenship, being over 18 years of age, and being resident in England for >10 years. The survey took ca. 30 minutes to complete and respondents were compensated for their time. Respondents who completed in < 15 minutes (*n* = 62, 2.8%) or > 24 hours (*n* = 14, 0.6%) and those submitting incomplete data (*n* = 35, 1.6%) were removed from the data set. The study design followed the British Psychological Society code of ethics.^12^ The final cohort size was 2,065 (45% females and 55% males) with a mean age of 47.8 ± 13.8 years (range 18–84 years). The majority of respondents considered themselves to live either in: suburbs (41%); a small town (20%); or a major town or city (19%); with the remainder in a village (16%) or in rural settings (4%). When summarized (80.8% urban and 19.3% rural), the distribution of the home environment compares favorably to the English average^13^ of 81.2% urban and 18.9% rural populations. When participants were asked about their employment status, the majority of respondents stated that they worked full time (*n* = 772) or were retired (*n* = 433). Only a small number of respondents (*n* = 188) identified themselves as currently unemployed. The participants were more likely than the English average to have been educated to degree level (41.4% compared to 30.8%) and much less likely to have gained qualifications below National Vocational Qualification (NVQ) level 1 (6.7% compared to 17.9%) or no qualifications at all (6.3% compared to 11.4%). The implied level of education may skew the resultant information.

The survey assessed respondents’ familiarity and personal experience of the 12 risks, their ease of understanding of the information provided, and their perceptions of the impact and likelihood of the risks. Responses were collected using Likert scales and slider bars. Personal experiences and respondents’ self‐rating of understanding were collected prior to the presentation of narrative statements on each risk, which contained three pieces of information about the adverse impacts of the risk. Where there was a high uncertainty in the supporting scientific evidence for specific impacts, this was stated. Statements were presented individually and followed by questions where participants were asked to self‐assess their understanding of the information provided and then indicate which organizations they believed were accountable for managing the residual risk in each case (government, scientists, industry, or self), and were then asked to consider impacts from the residual risks into the future. Additionally, respondents were asked to rate the severity of the impacts of the risk on the environment, on the economy, and on society. The presentational order of the risk information was randomized to minimize the effect this might have on the evaluations. The expert assessment of the risks has previously been described^7^ drawing from senior policy officials and technical policy developers within the United Kingdom.

Data were analyzed for descriptive statistics (mean and standard deviation), and the ranking of information was performed using SPSS software (SPSS Statistics, Version 20, IBM Corporation, Armonk, NY, USA). Additional tests used were Student's *t*‐test, a one‐way ANOVA with Tukey *post hoc* analysis (honest significant difference [HSD]), Pearson's correlation coefficient, and Kruskal–Wallis one‐way ANOVA for nonparametric data. While the use of parametric descriptive statistics and analysis (e.g., ANOVA) for ordinal data is open to controversy, the relatively large sample number and the Likert scales (with assumed equal intervals and formulated symmetrically) resulted in our choice to address them as parametric data.^14^ Statistical assessment allows for quantitative assessments of association, although we suggest that any inference of causal relationships between parameters should be treated with caution. ANOVA data are presented in standard format in accord with statistical practice.^15^, ^16^

## 3. RESULTS AND DISCUSSION

### 3.1. Personal Experience

**Figure 1:**
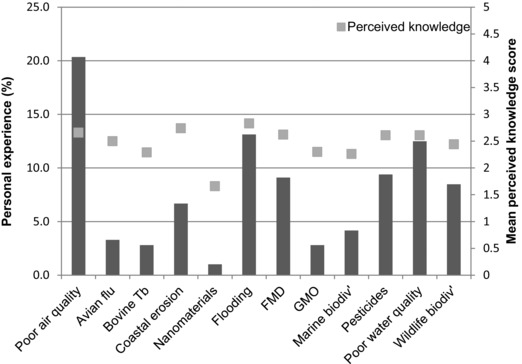
Plot showing public survey results (*n* = 2,065) identifying the incidence of personal experience (% of total population) and the mean perceived knowledge score (± standard deviation) of the 12 risk areas by the responders (*n* = 2,065; where perceived knowledge score was rated from 1 [“nothing at all”] to 5 [“very much”]). The personal experience scores were shown to be statistically significantly different using one‐way ANOVA (*F*(11,2054) = 119.90; *p*<0.005). Pearson correlation coefficient analysis showed that there was a statistically significant relationship between experience of the risk area and perceived knowledge score (*p*<0.01).

Survey participants (*n* = 2,065) first rated their personal experience of 12 individual policy risks. The majority of respondents had not experienced the risks studied (Fig. 1) but this varied across the presented risks, with a statistically significant difference between how much experience people had had with the 12 risks being identified using a one‐way ANOVA (*F*(11,2054) = 119.9; *p*<0.005). *Post hoc* comparisons using the Tukey HSD test ( Supporting Information A) indicated the most commonly experienced risks were those that expressed adverse impacts on air quality (20.3%) and through flooding (13.1%); the least being those associated with ENMs; (1%) and GMOs; (2.8%). Less than 10% of participants reported that they did not know whether or not they had experienced a risk, with the exception of nanomaterials and GMOs, where the percentages were 22% and 13%, respectively, an observation that may reflect the high technological component of these risks and a lack of awareness for their wide presence (for ENM, say) in consumer products. Participants’ perceptions of their prior knowledge for the 12 risks were shown to be significantly different for each risk across the studied group through a one‐way ANOVA (*F*(11,2054) = 208.2; *p*<0.005).

### 3.2. Perceived Knowledge

We next tested participants’ broader knowledge of the 12 risks, over and above their direct personal experience of them (Fig. 1). Risks where a broader knowledge was highest (Likert scale; 1 “nothing at all”; 5 “very much”; bars, Fig. 1) were flooding (2.83 ± 0.92), coastal erosion (2.73 ± 1.02), and air quality (2.67 ± 0.96). Nanomaterials (1.65 ± 0.93) attracted the lowest degree of broader perceived knowledge. Respondents’ broad knowledge was significantly correlated with their personal experiences of individual risks (*p*<0.01; Pearson's correlation and two‐tailed test; Fig. 1).

### 3.3. Comparative Rating of Risks

Next, brief narratives on the 12 risks were provided to participants, with the readability of narratives being assessed. (Narratives had a mean readability of > 4.5 on a Likert scale where 1 was “very difficult” to read, 3 “neither easy nor difficult,” and 7 “very easy” to read.) The narratives were used to present the uncertainty inherent to some risks (e.g., for nanomaterials, where dose–response relationships are a source of considerable uncertainty in their risk characterization) and enrich the expert assessment of the selected risks. Participants then rated the magnitude of the impacts that each risk posed to the environment, economy, and wider society, using slider bars with scales from “not at all serious” (score = 1) to “very serious” (score = 7; Table I). During this, all 12 risks were visible to participants concurrently, so relative comparisons, tradeoffs, and adjustments between the set of risks could be made by respondents in a “set” assessment, adjusting the slider bars for each to assemble an assessment of all 12 risks in concert. An ANOVA indicated that respondents did not equally value the impacts of each risk when the impacts were considered separately (environment *F*(11,2054) = 143.0; *p*<0.005; economy *F*(11,2054) = 167.6; *p*<0.005; and social *F*(11,2054) = 131.9; *p*<0.005) compared with when impacts were considered collectively as a set (*F*(11,2054) = 73.4; *p*<0.005) as shown in Tables S1 to S6, Supporting Information A.

For environmental impacts (Table I), risks with the highest scores either affected a public good, e.g., air quality (*p*<0.005, except marine and wildlife biodiversity, as described in Supporting Information C), water quality (*p*<0.05, except flooding, marine biodiversity, and pesticide use); or the risk posed a threat to a receptor representing a high stock at risk, e.g., the biodiversity of terrestrial or marine wildlife, (*p*<0.05, except water quality and air quality). The lowest environmental impact was expressed for well‐publicized risks, where an adverse environmental impact had been arguably less than expected (e.g., AI, BTb, and GMOs, *p*<0.05, except GMO compared to FMD).

**Table I:** Perceived Impact on the Environment, Economy, and Society Ranked by Mean Score (*n* = 2,065) and Standard Deviation Rated on a Seven‐Point Likert Scale

Impact on Environment		Impact on Economy		Impact on Society		Combined Impact	
Hazard Area	Score	Hazard Area	Score	Hazard Area	Score	Hazard Area	Score
Air quality	5.3 ± 1.6	Flooding	5.1 ± 1.5	Air quality	5.4 ± 1.5	Flooding	5.0
Wildlife biodiversity	5.2 ± 1.7	FMD	5.1 ± 1.6	Water quality	5.2 ± 1.7	Air quality	4.9
Marine biodiversity	5.2 ± 1.6	Bovine Tb	4.8 ± 1.7	Flooding	4.9 ± 1.5	Water quality	4.8
Water quality	5.0 ± 1.6	Marine biodiversity	4.4 ± 1.6	Nanomaterials	4.8 ± 1.6	FMD	4.6
Pesticides	4.9 ± 1.6	Water quality	4.2 ± 1.7	Pesticides	4.8 ± 1.6	Marine biodiversity	4.6
Flooding	4.9 ± 1.7	Avian influenza	4.2 ± 1.7	Avian influenza	4.7 ± 1.7	Pesticides	4.6
Nanomaterials	4.7 ± 1.7	Nanomaterials	4.0 ± 1.6	FMD	4.4 ± 1.6	Nanomaterials	4.5
Coastal erosion	4.6 ± 1.9	Air quality	4.0 ± 1.7	Bovine Tb	4.4 ± 1.7	Bovine Tb	4.5
FMD	4.4 ± 1.8	GMO	4.0 ± 1.6	GMO	4.4 ± 1.6	Wildlife biodiversity	4.4
GMO	4.3 ± 1.7	Pesticides	4.0 ± 1.6	Wildlife biodiversity	4.2 ± 1.7	Avian influenza	4.3
Bovine Tb	4.1 ± 1.8	Wildlife biodiversity	3.8 ± 1.7	Marine biodiversity	4.2 ± 1.6	GMO	4.2
Avian influenza	3.9 ± 1.8	Coastal erosion	3.8 ± 1.7	Coastal erosion	3.9 ± 1.7	Coastal erosion	4.1

Scale: 1: not at all serious; *2: a little serious; 3: somewhat serious; 4: moderately serious; 5: quite serious; 6: serious;* and 7: very serious. (Participants could not see the italic labels “a little serious, somewhat serious, moderately serious, quite serious and serious” but saw the gridlines. The categories are indicators for analysis.) The gray boxes highlight the same risk for each impact. For statistical analysis, see Supplementary Material.

For economic impacts, flooding and FMD were considered to pose the highest economic impacts (Table I). These remain high in the public consciousness and have resulted in a significant economic impact in the United Kingdom over the last 15 years. Costs for managing the residual national flood risk are estimated at ca. £1bn/annum, and a 2007 regional flooding event in England affected 55,000 properties, causing £3bn damage.^17^, ^18^ Recent FMD outbreaks cost £3bn (direct stock loss) plus £5bn (indirect costs such as loss of tourism).^19^ The lowest economic impact was scored for coastal erosion, possibly perceived as affecting only individual properties, which may account for its low ranking for social impact. Those risks ranked highest for societal impact affected a large human population and their health (air and water quality; Table I), while risks to terrestrial wildlife and marine biodiversity, potentially affecting numerous biota, were considered to have low social impacts.

### 3.4. Future Risks

**Figure 2:**
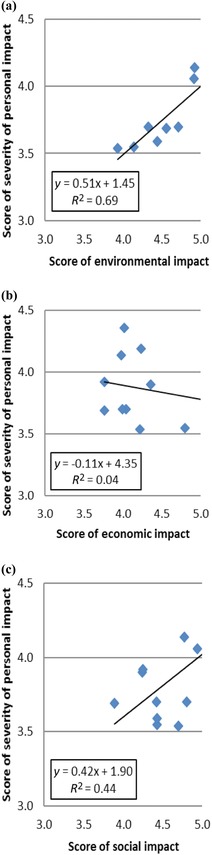
Plots showing correlation between the score of perceived personal impact for each risk and the score of perceived: (a) environmental impact, (b) economic impact, and (c) social impact for each risk with correlation (*R* ^2^) scores noted.

Respondents next considered how the 12 risks might evolve going forward––the severity of the personal impact for each risk over the next 12–18 months, compared to a combined assessment (Table II). Analysis of the combined assessment suggested no statistically significant differences between the risks (*F*(11,2054) = 73.4; *p*>0.05). However, analysis of the personal impact over the next 12–18 months suggested differences (*F*(11,2054) = 33.3; *p*<0.05). There is some indication that an assessment of severity for personal impact is related to a respondent's personal experience of the risks (with a similar order in the highest scored hazard areas; *r* = –0.015, *p*<0.05, two‐tailed Pearson correlation), yet it was not possible to identify further relationships. When examining perceived personal impacts (Table II), there are clusters of hazards that might be linked to risk perception paradigms.^20^ The lowest ranked hazards are animal diseases unlikely to have significant impacts on individuals who do not live in or draw their livelihood from rural environments. Low levels of correlation were observed (Fig. 2) when the perceived personal impact was compared to the scores of environmental, economic, and social impacts for each risk. The relationship between perceived personal impact and economic impact was unclear, and reinforces the challenges faced by government economists in trying to apply a value to public goods.

**Table II:** Comparing Total Severity of the Perceived Individual Impact (Over Next 12–18 Months; Seven‐Point Scale), Likelihood of Personal Affect (Over Next 12–18 Months; Seven‐Point Scale), and Combined Ratings (Seven‐Point Scale) Taken from an Assessment of Environmental, Social, and Economic Attributes

	Severity of Impact	Likelihood	Combined
Air quality	4.4	2.6	4.9
Water quality	4.2	2.3	4.8
Pesticides	4.1	1.9	4.6
Flooding	4.1	2.5	5.0
Marine biodiversity	3.9	1.9	4.6
Nanomaterials	3.9	2.8	4.5
GMO	3.7	1.8	4.2
Coastal erosion	3.7	2.4	4.1
FMD	3.6	2.3	4.6
Bovine Tb	3.6	2.0	4.5
Avian influenza	3.5	2.0	4.3

**Figure 3:**
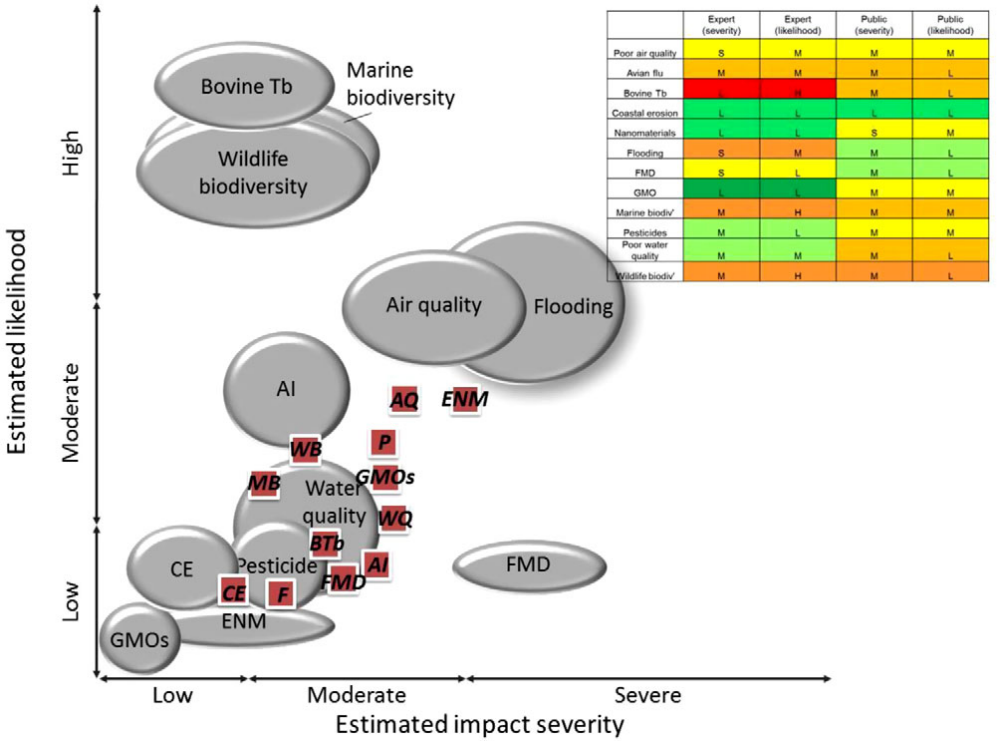
An illustrative appraisal of 12 strategic environmental risks for Defra representing both the expert assessment (ellipses) and public assessment collected during this study (boxes) where inserted table shows color scale representation of figure (color visible in on‐line version). Ellipses reflect the relative magnitude and two‐dimensional uncertainty in likelihood and consequence for residual policy risks, assessed over a 12–18‐month horizon (from autumn 2011) assuming existing risk management measures in place. Their positions are informed through a flow of supporting evidence, independent analysis, and deliberative process, whereas the public assessment of the risks is mean value (*n* = 2,065). AI is avian influenza, AQ is air quality, CE is coastal erosion, ENM is engineered nanomaterial, F is flooding, FMD is foot and mouth disease, GMO is genetically modified organism, MB is loss of marine biodiversity, WB is loss of wildlife biodiversity, and WQ is water quality.

We next employed the data from a comparative public assessment of the 12 policy risks (severity and likelihood over the next 12–18 months) and compared this (Fig. 3) with a prior expert analysis.^7^ Due to limitations in the collection of expert analysis, it is not possible to provide more than a subjective assessment between the two data sources. There are similarities between the expert and public assessments for coastal erosion, water quality, and air quality, but the public showed either lower assessments of likelihood (e.g., for wildlife and marine biodiversity, AI, and BTb) or of impact severity (e.g., FMD), where it may be argued that there is less personal experience of these risks, but greater awareness of the impacts (Fig. 3). For novel risks, such as GMOs and ENMs, participants expressed a higher assessment of impact severity compared to the experts, which may be related to a lack of familiarity with such risks.^15^ A follow‐up laboratory study of 109 survey participants deployed its set analysis to apportion a fictitious departmental budget between risks with recommendations for relative investments in public risk management.^21^

### 3.5. Perceptions of Responsibility

**Figure 4:**
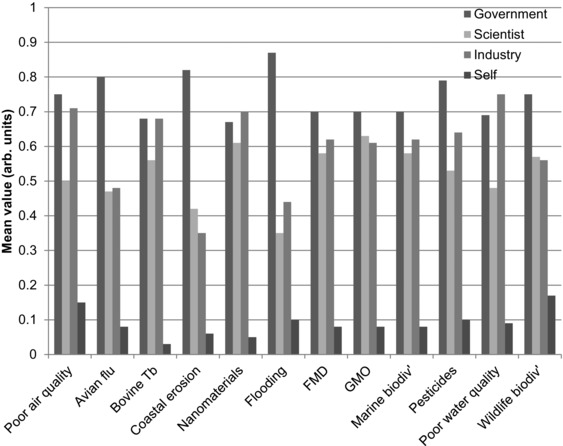
Mean value of perceived responsibility for risk management across the stakeholder groups identifying where responsibility lies (no responsibility = 0 and responsibility = 1).

The challenges of public risk management under resource constraints are considerable. Where should governments invest additional resources, across a risk portfolio, so to keep residual risks at a tolerable level on behalf of the public? As state funding becomes pressed, many governments are considering afresh the distribution of responsibilities between the various existing actors (and new ones) that deliver public risk management––for existing flood risk management and emerging concerns surrounding farm biosecurity, for example.^22^ Data from this survey make clear where study participants felt responsibility should lie for the 12 risks examined (Fig. 4), with the respondents rating self‐responsibility continuously lower than that of government, industry, or scientists. When these preferences were explored in detail (during a subsequent laboratory study),^21^ respondents perceived the government held the highest level of responsibility for the management of the 12 policy risks evaluated, followed by scientists and then industry, with personal responsibility reported to be lowest (data not presented here). The promotion of greater self‐responsibility for the management of public risks, for example, for flood risk by the Environment Agency, seems at odds with the assessment and thus acceptance of personal responsibility here (Fig. 4) and so further research into the acceptance of responsibilities for managing risks is required as government reevaluates opportunities for risk and cost sharing between actors.^23^ While recent research suggests advances in risk communication have occurred,^24^, ^25^ these mostly relate to single environmental risks, with advances attributed to the recognition, in revised communication tools, of specific risk characteristics.

Managing a portfolio of policy risk is clearly a different proposition than managing a single risk, and when groups of risks clustered by similar characteristics are considered, this complicates the communication of collective decisions and the attending rationale for resource management. We might usefully reflect on how the position with public strategic analysis of this sort has developed since earlier incarnations of this were deployed in the United States and elsewhere.^3^, ^5^ The authors believe that there is enhanced public recognition of the balancing requirements that governments and their agencies make on environmental policy risk and, due to a helpful debate on, and communication of, the multidimensional characteristics of environmental harm, a richer understanding of how such risks are expressed and longer‐term features of the damage that might ensue. This said, citizens’ personal experiences remain fundamental to their individual response and engagement with policy‐level risk.

## 4. CONCLUSIONS

In concluding, this study supports the theory that personal experience affects the knowledge and assessment of individual risk. Where scores of personal experience were high in the study population, they were positively correlated with perceived knowledge scores. Providing information to citizens supports perceptions of policy risk, but if the public has difficulty in understanding the information, or there are gross uncertainties in the information provided, this could lead to reduced engagement. This was noted for ENMs, as highlighted by the level of uncertainty within this field. This relationship should be investigated further, given the complex character of environmental harms.^1^, ^2^, ^3^, ^4^, ^5^, ^6^, ^7^ Environment, social, and economic impacts within an overall assessment of 12 risks were considered. Citizens can distinguish between these classes of impact and provide separate (and informed) assessments of each, with the exception of the economic attribute (Fig. 3), which does not seem to reflect the overall severity of the risks. We note that the responsibly of government to manage residual public risk was identified by respondents, with less accountability being ascribed to other stakeholders (scientists, individuals, and industry). Previous studies^26^ have shown disconnect between public perceptions of, and actual responsibility for, risks, suggesting the need for clarity and a well‐reasoned rationale about shared responsibilities for risk management, the apportioning of these responsibilities between actors, and the uptake of accountabilities by newly identified risk managers.

Supporting information showing statistical analysis is provided in Supporting Information A, Tables S1–S6.

## Supporting information


**Table S1**. Statistical analysis (ANOVA with Tukey HSD *post hoc*) showing variation between respondents personally affected by environmental risks (where * = *p*≤0.05; *** = *p*≤0.005). It is noticeable that personal experience of loss of wildlife biodiversity seems to be linked with experience of avian influenza, bovine Tb, pesticide use, and coastal erosion.
**Table S2**. Statistical analysis (ANOVA with Tukey HSD *post hoc*) showing variation between respondents' self‐assessment of personal knowledge of environmental risks (where * = *p*≤0.05; ** = *p*≤0.01; and *** = *p*≤0.005).
**Table S3**. Statistical analysis (ANOVA with Tukey HSD *post hoc*) showing variation between respondents' self‐assessment of environmental impact of environmental risks (where * = *p*≤0.05; ** = *p*≤0.01; and *** = *p*≤0.005).
**Table S4**. Statistical analysis (ANOVA with Tukey HSD *post hoc*) showing variation between respondents' self‐assessment of economic impact of environmental risks (where * = *p*≤0.05; ** = *p*≤0.01; and *** = *p*≤0.005).
**Table S5**. Statistical analysis (ANOVA with Tukey HSD *post hoc*) showing variation between respondents' self‐assessment of social impact of environmental risks (where * = *p*≤0.05; ** = *p*≤0.01; and *** = *p*≤0.005).
**Table S6**. Statistical analysis (ANOVA with Tukey HSD *post hoc*) showing variation between respondents' self‐assessment of combined impact of environmental risks (where * = *p*≤0.05; ** = *p*≤0.01; and *** = *p*≤0.005).
**Supporting Information B**. Questionnaire information presented to respondents.
**Supporting Information C**. risk information presented to respondents.
