# Supplementary material for: Engaging with Comparative Risk Appraisals: Public Views on Policy Priorities for Environmental Risk Governance
Source: Risk Anal. 2017 Mar 17;37(9):1683–92. doi: 10.1111/risa.12735 (PMC6849548; doi:10.1111/risa.12735)
Supplement: Supplementary file 1 — Table S1. Statistical analysis (ANOVA with Tukey HSD post hoc) showing variation between respondents personally affected by environmental risks (where * = p≤0.05; *** = p≤0.005). It is noticeable that personal experience of loss of wildlife biodiversity seems to be linked with experience of avian influenza, bovine Tb, pesticide use, and coastal erosion. Table S2. Statistical analysis (ANOVA with Tukey HSD post hoc) showing variation between respondents' self‐assessment of personal knowledge of environmental risks (where * = p≤0.05; ** = p≤0.01; and *** = p≤0.005). Table S3. Statistical analysis (ANOVA with Tukey HSD post hoc) showing variation between respondents' self‐assessment of environmental impact of environmental risks (where * = p≤0.05; ** = p≤0.01; and *** = p≤0.005). Table S4. Statistical analysis (ANOVA with Tukey HSD post hoc) showing variation between respondents' self‐assessment of economic impact of environmental risks (where * = p≤0.05; ** = p≤0.01; and *** = p≤0.005). Table S5. Statistical analysis (ANOVA with Tukey HSD post hoc) showing variation between respondents' self‐assessment of social impact of environmental risks (where * = p≤0.05; ** = p≤0.01; and *** = p≤0.005). Table S6. Statistical analysis (ANOVA with Tukey HSD post hoc) showing variation between respondents' self‐assessment of combined impact of environmental risks (where * = p≤0.05; ** = p≤0.01; and *** = p≤0.005). Supporting Information B. Questionnaire information presented to respondents. Supporting Information C. risk information presented to respondents. [file RISA-37-1683-s001.doc]

**SUPPORTING INFORMATION**

**Engaging with comparative risk appraisals – public views on policy priorities for environmental risk governance**

SUPPLEMENTARY A: TABLES SHOWING STATISTICAL ANALYSIS

Table S1. Statistical analysis (ANOVA with Tukey HSD *post hoc*) showing variation between respondents personally affected by environmental risks (where * = p≤0.05; *** = p≤0.005). It is noticeable that personal experience of loss of wildlife biodiversity seems to be linked with experience of avian influenza, bovine Tb, pesticide use and coastal erosion.


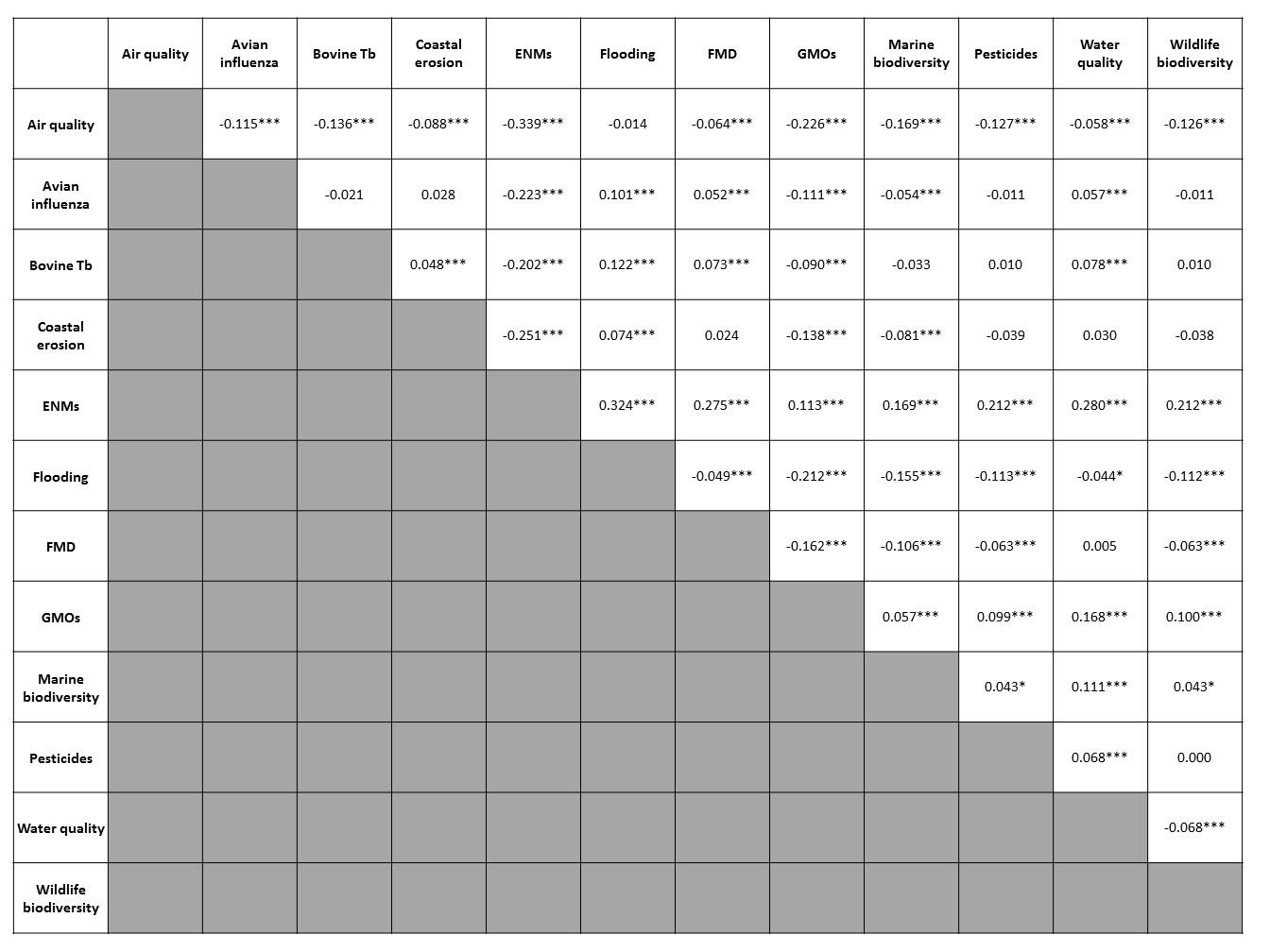


Table S2. Statistical analysis (ANOVA with Tukey HSD *post hoc*) showing variation between respondents self-assessment of personal knowledge of environmental risks (where * = p≤0.05; ** = p≤0.01; and *** = p≤0.005).

**
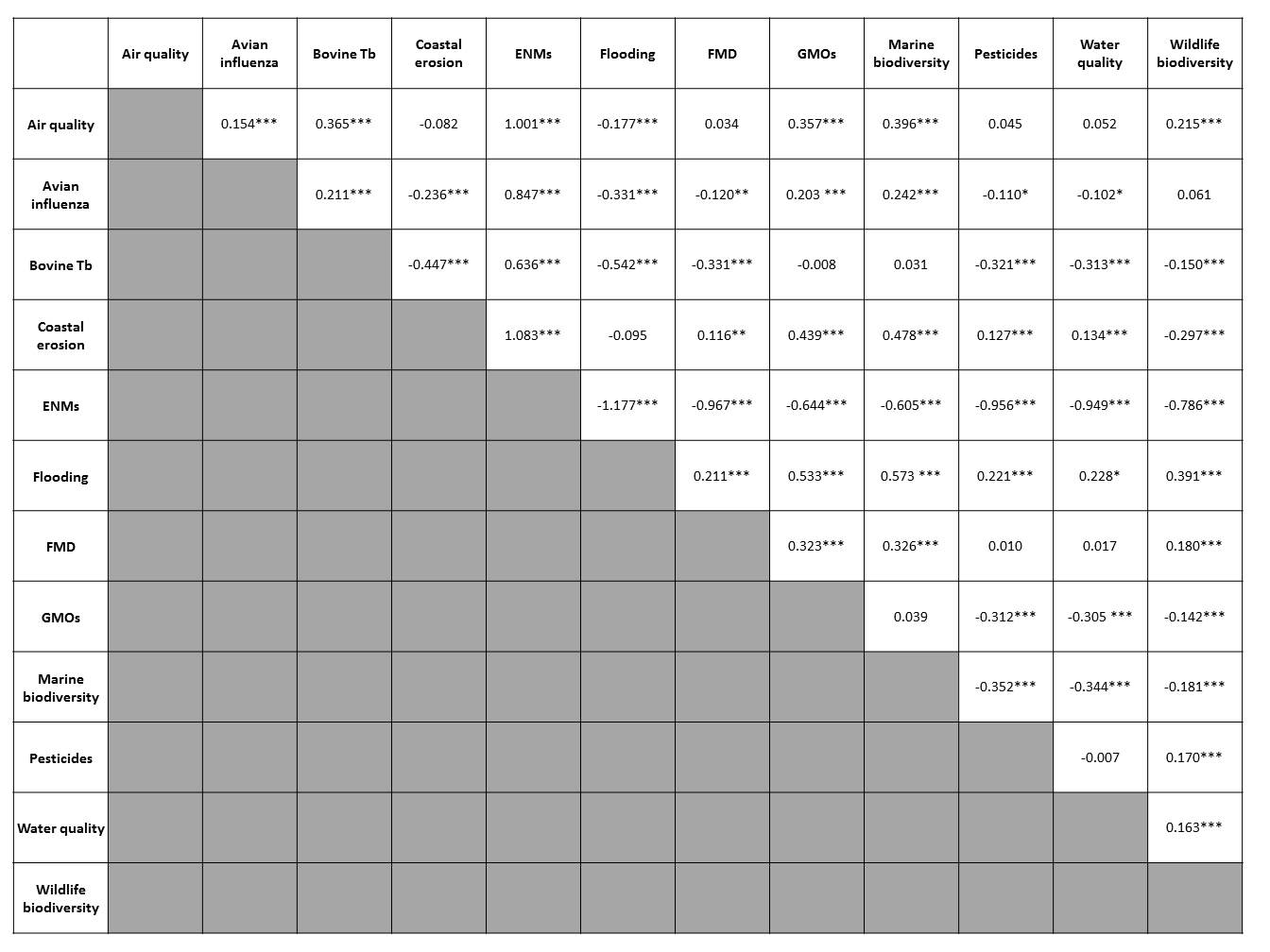
**

Table S3. Statistical analysis (ANOVA with Tukey HSD *post hoc*) showing variation between respondents self-assessment of environmental impact of environmental risks (where * = p≤0.05; ** = p≤0.01; and *** = p≤0.005).

**
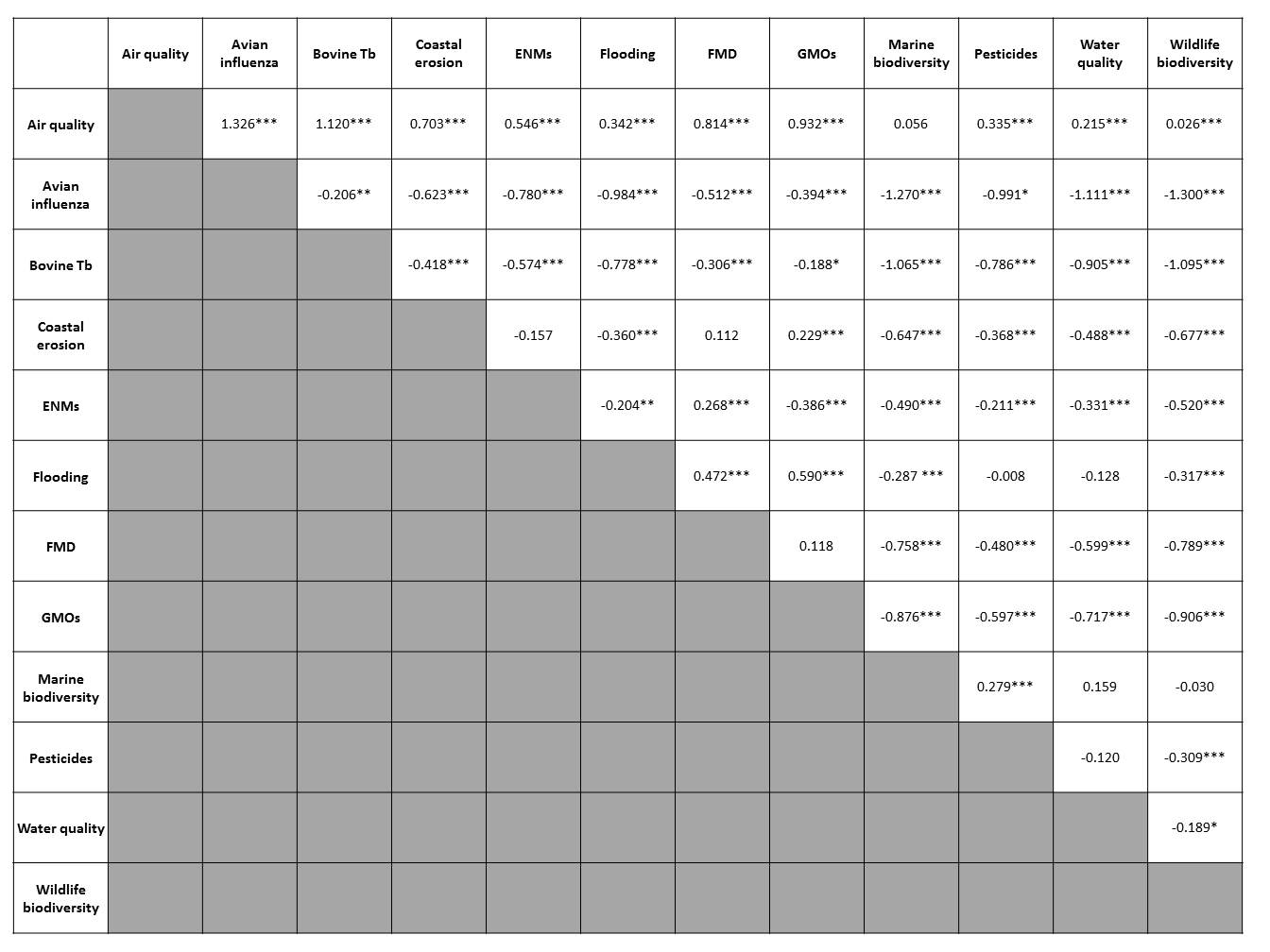
**

Table S4. Statistical analysis (ANOVA with Tukey HSD *post hoc*) showing variation between respondents self-assessment of economic impact of environmental risks (where * = p≤0.05; ** = p≤0.01; and *** = p≤0.005).

**
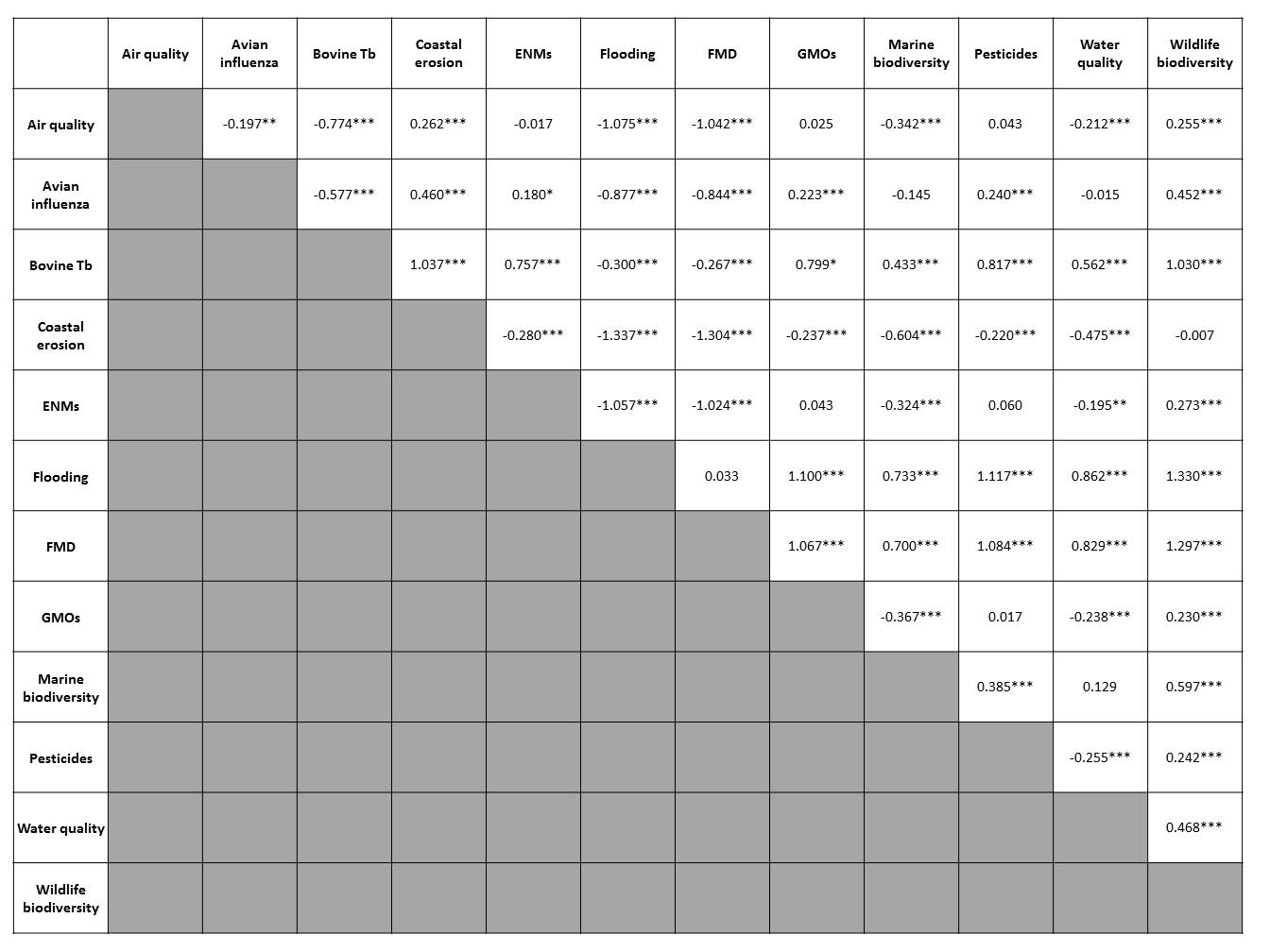
**

Table S5 – Statistical analysis (ANOVA with Tukey HSD *post hoc*) showing variation between respondents self-assessment of social impact of environmental risks (where * = p≤0.05; ** = p≤0.01; and *** = p≤0.005).

**
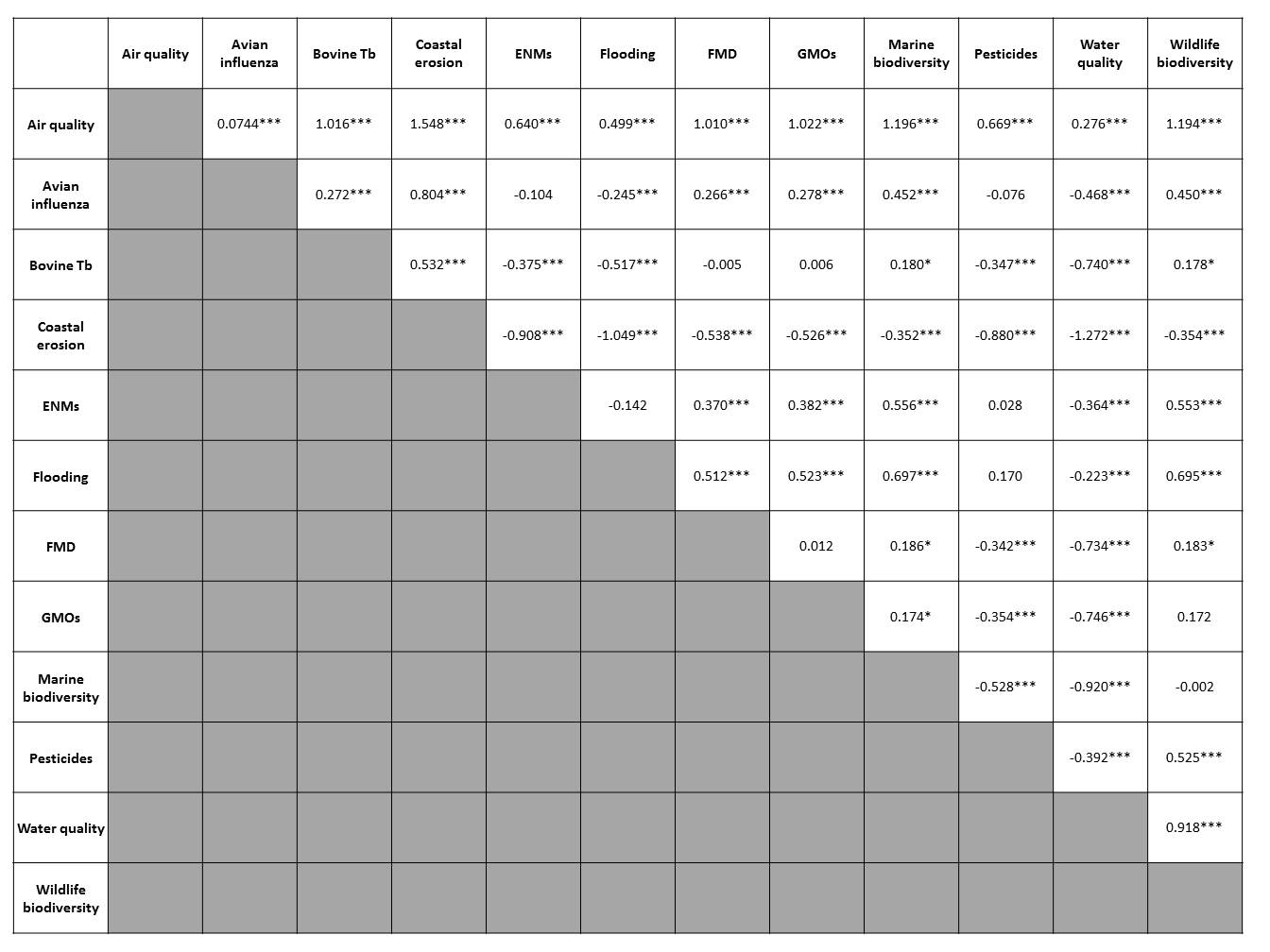
**

Table S6 – Statistical analysis (ANOVA with Tukey HSD *post hoc*) showing variation between respondents self-assessment of combined impact of environmental risks (where * = p≤0.05; ** = p≤0.01; and *** = p≤0.005).

**
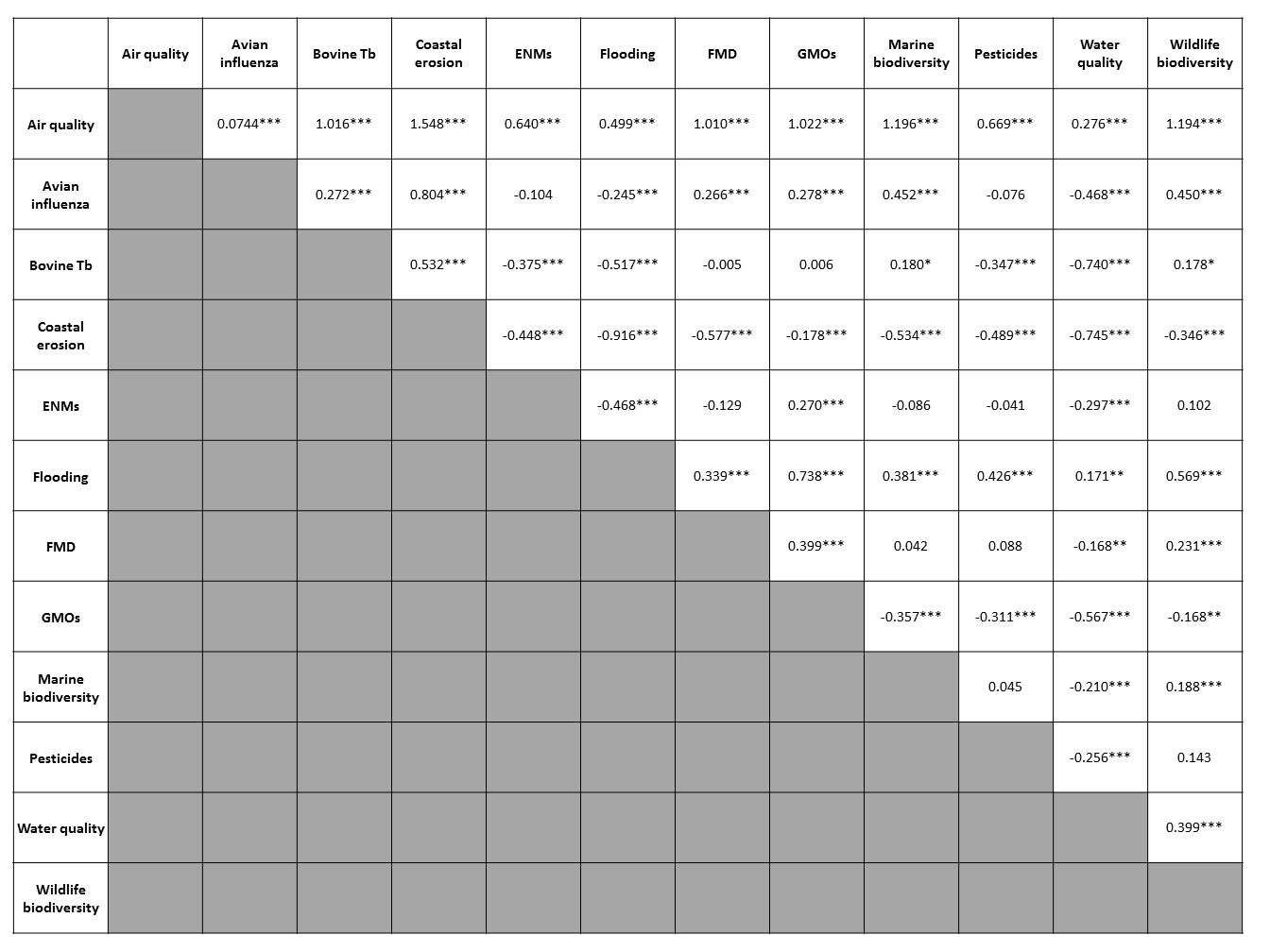
**

SUPPLEMENTARY B: QUESTIONNAIRE INFORMATION PRESENTED TO RESPONDENTS

A web-based survey was developed to determine the measure the public views on policy priorities within the environmental sector. Each question (unless specified) addressed the full range of environmental hazards previously identified (namely : poor air quality; the risk of an avian influenza (AI) incursion; the accelerated spread of bovine tuberculosis (Bovine TB); risks from coastal erosion; the risk of regional scale flooding; the risk of a foot and mouth disease (FMD) incursion; of exposure to genetically modified organisms (GMOs); the loss of marine biodiversity; exposures to engineered nanomaterials (ENM); of human health effects from pesticides; the risk of a derogation of water quality; and the risk of a loss of wildlife biodiversity). The survey presented questions as follows.

1. “Please tell us in the next section about your experience and knowledge about the following environmental issues.”

2. “How much do you know about the following issues?  Please rate your knowledge on the scale next to each item.” Each of the 12 hazards was presented in a randomised order, with a response range was from 1 'Nothing at all' to 5 'Very much'.

3. “Have you been personally affected by [the hazard]”: response range was 1 'Yes', 2 'No'; 3 'Don't know'.

3a. If the answer was yes, a follow up question was “When did you last personally experience [the hazard]:” followed by a list of the 12 hazards. The response range was 1 'Less than 1 year ago', 2 'In the last 1 to 3 years', 3 'In the last 4 to 7 years', 4 'In the last 8 to 10 years', 5 'More than 10 years ago'.

Next, information about a hazard was presented and the information provided on each hazard is presented in the following section. The ordering of hazards was randomised. For example, in the case of air quality:

*“Poor air quality arises from the introduction of gases or particles to the air, causing harm or discomfort to humans and/or the environment – often in the form of an on-going, daily exposure. The main man-made sources of air pollution are industry, power generation and vehicle emissions. People, animals and plants will all be affected by poor air quality. There has been extensive research on the impacts on human health with current levels of particulate pollution estimated to reduce average life expectancy in the UK by approximately 6 months.”*

Following this, participants were asked:

4. “Understanding this information was:” 1 'Very difficult', 2 ' Difficult', 3 'Somewhat difficult', 4 'Neither easy nor difficult', 5 'Somewhat easy', 6 'Easy', 7 'Very easy'.

5. “Next we would like to know who you think is responsible for dealing with poor air quality. Please tick all that apply”. Responsibility was assessed for: 'research to research air quality'; 'responsibility to inform the public about air quality'; 'responsibility to manage air quality'; 'responsibility to take action on poor air quality'. Participants ticked perceived applicability to: 'government', 'scientists', 'industry', 'me'.

6. “Next we would like to know the levels of trust you have in those who are involved in dealing with poor air quality”: 'I trust the government to make the right decisions on air quality'; 'I trust scientists to make the right research decisions on air quality'; 'I trust industry and business to make the right decisions on air quality'. The response range was 1 'Strongly disagree', 2 'Disagree', 3 'Disagree somewhat', 4 'Neither agree nor disagree', 5 'Agree somewhat', 6 'Agree', 7 'Strongly agree'.

7. “We would now like to find out what you think about the likelihood and impact of poor air quality. The likelihood of poor air quality affecting me in the next 12-18 months is:” 1 'Very low', 2 'Low', 3 'Moderate', 4 'High', 5 'Very high' 6 'Unable to judge'.

8. “If poor air quality affects me in the next 12-18 months, the impact is likely to be:” 1 'Very low', 2 'Low', 3 'Moderate', 4 'High', 5 'Very high' 6 'Unable to judge'.

9. “Do you think that risks associated with air quality will change in the next 5-10 years? “1 'Risks will reduce a lot', 2 'Risks will reduce', 3 'Risks will reduce somewhat', 4 'Risks will stay the same', 5 'Risks will increase somewhat', 6 'Risks will increase', 7 'Risks will increase a lot'.

10. “Please rate the extent of the impact that poor air quality has on the environment; the economy; human health and society on the scale below.” 1 'Not at all serious' to 7 'Very serious'.

Questions 4-10 were repeated for each of the 12 hazards. The order of presentation was randomised.

The next section presented the 12 hazards altogether in a table, each with a slider bar. There were two tables:

11. “Please indicate the level of likelihood that you personally will be affected by these environmental issues in the next 12-18 months.  Move the slider bars below”. 1 'Not at all likely' to 7 'Very likely'.

12. “Please indicate the level of severity of risks associated with each environmental issue using the slider bars below.” 1 'Not at all severe' to 7 'Very severe'.

13. “The next question will ask you about your ideas regarding investment into the environmental issues which were presented in this questionnaire.” The 12 hazards were presented and for each hazard participants were given a dichotomous choice: 'Tick here if you would like more investment and the potential for decreased risks' or ' Tick here if you would be prepared to accept less investment and the potential for increased risks'.

Demographic information was then collected using the following questions:

14. “Are you:” 1 'Female', 2 'Male'.

16. “What is your home environment?” 1 'Urban (major town or city centre)', 2 'Suburban (town or city outskirts)', 3 'Small town', 4 'Village or hamlet', 5 'Rural'.

17. “Which county do you live in? Please select from the dropdown menu” [list of counties]

18. “What is the highest educational qualification that you hold, including any that you have gained since leaving school?” 1 'Degree or degree equivalent, and above', 2 ' Other higher education below degree level', 3 ' A levels, vocational level 3 & equivalents', 4 ' GCSE/O Level Grade A*-C, NVQ level 2 & equivalents', 5 'Qualifications at NVQ level 1 and below', 6 ' Other qualifications', 7 'No qualifications').

19. “Does your household own or rent the accommodation you live in? 1. Owns outright, 2. Owns with a mortgage or loan, 3. Part owns and part rents (shared ownership), 4. Rents (with or without housing benefit), 5. Lives rent free.

20. Do you have any children of school age or younger? 1. Yes, 2. No

21. Do you have caring responsibilities for any family members other than children? 1. Yes, 2. No

22. “To which job category do you belong? If you have more than one job, please answer for your main job or the one at which you spend the most time. Please select from the dropdown menu” 1. Full time education, 2 full time housewife/husband, 3 part-time employed, 4 full time employed, 5 self-employed, 6 currently unemployed, 7 retired, 8 other.

22. Which one of these describes the type of work you do in your current job? Please select from the dropdown menu 1 'Professional' 2 'Associated professional and technical' 3 'Administrative and secretarial' 4 'Manager or senior official' 5 'Personal services', 6 'Retail and customer service' 7 'Skilled trades' 8 'Process plant or machine operator' 9 'Elementary occupations' 10 'Other'.

21. Which sector do you work in? If you have more than one job, please answer for your main job or the one at which spend the most time: [a range of jobs were presented].

22. On average, how much do you get paid before tax and other deductions are removed? Please select from the dropdown menu [range: from less than 20,000 per year to more than 100,000 per year with increments of 20,000].

23. How would you describe your ethnic group? [Ethnic groups were presented].

SUPPLEMENTARY C: RISK INFORMATION PRESENTED TO RESPONDENTS

**Coastal erosion**

Natural weathering processes - waves, tides, currents and storm surges - constantly affect the English coastline, causing erosion. The coastlines that are most affected are those on the east and south of the country, and those around the Isle of Wight. Erosion is either gradual or drastic (e.g. cliff slump). Local authorities estimate that 200 properties may be lost over the next 20 years, with approximately 2,000 vulnerable properties in England. The threat of coastal erosion reduces property values and has a detrimental impact on individual and community well-being. Coastal erosion does provide natural benefits to beaches and habitat.

**Nanomaterials**

Nanomaterials are tiny man-made particles, with one dimension less than 100 nanometres. If they are routinely or accidentally released (from manufacturing facilities) or if they are present in products that have reached the end of their lives, they may affect the health of workers, the general public or the wider environment. In some instances, nanomaterials may change into new compounds that could be hazardous. The long-term effects of nanomaterials are currently very uncertain, because of the lack of data.  However, evidence is emerging of effects in fish, with some nanomaterials able to cross the blood-organ barrier. The toxicity of constituent compounds (e.g. cadmium) in nanomaterials may lead to environmental and health harm. The deliberate addition of nanomaterials to soil or water, in ignorance of the potential consequences, may lead to the loss of soil fertility or reduced water quality. Once released, nanomaterials cannot be recovered, and the impacts are likely to be latent.

**Foot and Mouth Disease**

Foot and Mouth Disease is a highly contagious viral disease that affects cows, pigs, goats, sheep and deer. It can spread rapidly via many routes. The most critical factors that determine the size of an outbreak are the time to detection and the numbers of livestock in the area where disease occurs. Control of this disease is normally achieved by killing infected animals and dangerous contacts.  Vaccines are available and they can be very effective, if the vaccine strain used is a good match to the field virus.  No single vaccine is effective against all types of the disease.

**Flooding**

Floods are a natural process that may occur anytime throughout the year, originating from rivers, the sea, rainfall or rising groundwater. The scale of flooding differs depending upon a combination of weather, rainfall patterns, topography and the degree of development (urbanisation, sewer capacity) in an area. The severity of a flood depends on the speed and duration of inundation, the numbers of people in the area and the value of the property and infrastructure affected. Flooding from the sea is considered more severe than flooding from rivers, and approximately 50% of the total properties at risk of flooding in England and Wales are located along the coast.

**GMOs**

Genetically modified organisms (GMOs) are those in which the genetic material has been altered in a way that does not occur naturally by mating and/or natural recombination. In the last 5 years only genetically modified (GM) pest-resistant potatoes have been grown in research trials in the UK. Research trials involving GM microorganisms have mainly involved GM bacteria for use as vaccines. All research trials involving live GMOs that are to be exposed to the environment must be authorised by government, and the risks to human health and the environment must be assessed. There is currently no commercial cultivation of GM crops in the UK.  GM crops are grown for commercial purposes elsewhere in Europe, particularly Spain (maize) and Germany (potatoes). GM animal feed is used in the UK.  It is mostly imported, which brings the risk of accidental spillage or release.

**Marine Biodiversity**

Reduced marine biodiversity arises from a reduction in the abundance, variety or complexity of the UK’s marine biological diversity. Drivers of marine biodiversity loss include human activities (such as intensive fishing, which results in habitat destruction), the spread of invasive species and changing climate. The impacts of marine biodiversity loss are very complex to understand and predict. Changes in marine biodiversity can be progressive or rapid, but are usually measured in years and decades rather than months.

**Pesticides**

Pesticides used as plant protection products include insecticides, herbicides and fungicides. Exposure to these substances at excessive levels can lead to acute and chronic toxicity to humans (either when pesticides are being applied or when pesticide-treated food is consumed), domestic animals, wildlife and plants. Pesticides are widely used by farmers to provide a quality and consistency of control on pests, weeds and diseases, thereby helping to produce high quality, reasonably priced and locally grown foodstuffs.

**Water Quality**

In England and Wales there are approximately 6,000 water bodies (e.g. rivers, lakes, estuaries), 500 bathing water sites and 125 shellfish sites, as well as groundwater bodies. Pressures affecting water quality may derive from episodic events (e.g. storm-related sewage overflows or chemical spills) or chronic events (e.g. nutrient run-off from agricultural land and water abstraction). In England, approximately 30% of surface water and 60% of groundwater bodies are at good or better ecological status. Unsustainable water abstraction may be contributing to environmental damage at up to 500 sites in England. At least 50% of surface waters are affected by diffuse nutrient pollution originating from the agricultural and transport sectors.

**Wildlife Biodiversity**

Reduced wildlife biodiversity arises from a reduction in the abundance, variety or complexity of the UK’s wildlife biodiversity. The impact of any change in biodiversity is difficult to quantify - there are complex interactions with human wellbeing, there is a relatively poor understanding of ecosystem complexity and there is general scarcity of data. In the UK, the general trend is towards a loss in biodiversity, mostly as a result of habitat loss (due to land use change), pollution, invasive species and changing climate. Over time, incremental damage may have cumulative effects and lead to significant, irreversible harm with knock-on impacts.

**Air Quality**

Poor air quality arises from the introduction of gases or particles to the air, causing harm or discomfort to humans and/or the environment – often in the form of an ongoing, daily exposure. The main man-made sources of air pollution are industry, power generation and vehicle emissions. People, animals and plants will all be affected by poor air quality. There has been extensive research on the impacts on human health with current levels of particulate pollution estimated to reduce average life expectancy in the UK by approximately 6 months.

**Bird Flu**

Bird flu (or Avian influenza) is caused by the Influenza A viruses, and is most common in wild bird populations (e.g. waterfowl). Contact with wild birds represents the most common transmission route of bird flu to domestic poultry, and the occurrence and geographical distribution of bird flu can be linked to seasonal migration routes of wild birds. If the virus mutates to a highly pathogenic form then infected poultry farms can experience very high mortality rates. Mutation to the highly pathogenic variety is of concern because it may increase the potential for cross-species infection (e.g. to pigs and humans). Worldwide, 519 cases of bird flu in humans and 309 deaths have been reported since 2003, but transfer to humans is not common, often requiring very close contact with infected birds.

**TB in Cattle**

TB in cattle (or bovine tuberculosis) is a bacterial disease, with cattle as the primary host in England. It is a “notifiable” disease because, in rare instances, it can be transmitted from animals to humans. It is currently found in cattle herds across England, mostly in central and south-western regions. Wildlife (particularly badgers and deer) are known to act as reservoirs for the disease. The incidence of bovine TB in England is increasing.  In 2009, 25,000 cattle were slaughtered, resulting in government compensation of £31 million and an overall cost to the taxpayer of about £90m.
